# Dataset of knowledge, attitude, practices and psychological implications of healthcare workers in Pakistan during COVID-19 pandemic

**DOI:** 10.1016/j.dib.2020.106234

**Published:** 2020-09-01

**Authors:** Muhammad Qasim, Usman Ayub Awan, Muhammad Sohail Afzal, Muhammad Arif Nadeem Saqib, Shajee Siddiqui, Haroon Ahmed

**Affiliations:** a Department of Economics, Finance and Statistics, Jönköping University, Jönköping, Sweden; b Department of Medical Laboratory Technology, University of Haripur, Haripur, Khyber Pakhtunkhwa (22620), Pakistan; c Pakistan Health Research Council, Head Office, Islamabad, Pakistan; d Department of Medicine, Pakistan Institute of Medical Sciences, Islamabad; e Department of Biosciences, COMSATS University Islamabad (CUI), Islamabad, Pakistan

**Keywords:** COVID-19 Pandemic, Healthcare workers, Psychological effects, Knowledge, COVID-19 infection prevention, Pakistan

## Abstract

The COVID-19 pandemic has created a global health emergency and has a huge impact on the health care workers, especially on their mental health. The dataset presented was an assessment of COVID-19 related knowledge, attitude, practices and its effects on the mental health of frontline healthcare workers in Pakistan. The data were collected using a snowball sampling technique. A questionnaire was developed assessing sociodemographic characteristics (6 items), knowledge (11 items), attitude (5 items), practices (6 items), information sources (1 item) and psychological implications (12 items) and distributed using online tools. The dataset includes 476 healthcare workers in Pakistan. The dataset will help to prevent and curb the spread of COVID-19 among health workers and contribute to policymakers. Furthermore, our dataset provides detailed insights into different risk factors of psychological problems, and it may be served as the reference for various in-depth surveys.

## Specifications Table

**Table:** 

Subject	Public health
Specific subject area	Health psychology, Infectious Diseases
Type of data	Primary data, Figure, Tables
How data were acquired	Data were collected using questionnaire based online survey (Google forms) and converted into the .xlsx format. A copy of the survey is incorporated and available online on following link https://docs.google.com/forms/d/e/1FAIpQLScP_mf4b6exnDrQDs2Y1Qg7CuyLxzcnf5NzWhyIkl2SycnRzA/viewform?usp=sf_link
Data format	Raw, Analyzed, Filtered (Descriptive statistics)
Parameters for data collection	The survey dataset was collected from 476 healthcare workers through online tools who were dealing with patients during COVID-19 pandemic.
Description of data collection	A combination of purposive and snowball methods was used to select the healthcare workers respondents, via WhatsApp, Facebook, Twitter and Email. The online questionnaire was sent to those healthcare workers who were dealing the COVID-19 patients directly or indirectly.
Data source location	Region: Asia Country: Pakistan
Data accessibility	Mendeley data Direct URL: https://data.mendeley.com/datasets/j6mxjvwsgk/draft?a=1881f5d6-0938-4f30-960a-1f7fd05deb76

## Value of the Data

• The current dataset provides information about the knowledge, attitude and practices of health care workers towards COVID-19. It also describes the significant risk factors associated with mental health issues of healthcare workers due to COVID-19.
• All researchers, psychological experts/institutes, public health experts/institutes, and policymakers can get benefit from our data to understand the essential knowledge and compliance with safe practices of healthcare workers from Pakistan. The data can also support other countries around the world to understand the mental health challenges of healthcare workers during COVID-19 pandemic.
• The data will be valuable for the researchers who want to determine the association between COVID-19 related knowledge, attitude and practices of healthcare workers and the onset of psychological disorders. This dataset can be utilized by other researchers to apply statistical modelling for identifying characteristics that could bring new perceptions about the pandemic and how to fight it.
• Analysis of dataset is beneficial for the policymakers and hospital administrations to evaluate the primary cause of psychological illnesses among healthcare workers, thus helping to plan preventive strategies. All institutions involved in public health and infection control can benefit from these data.

## 1 Data Description

**Fig. 1:**
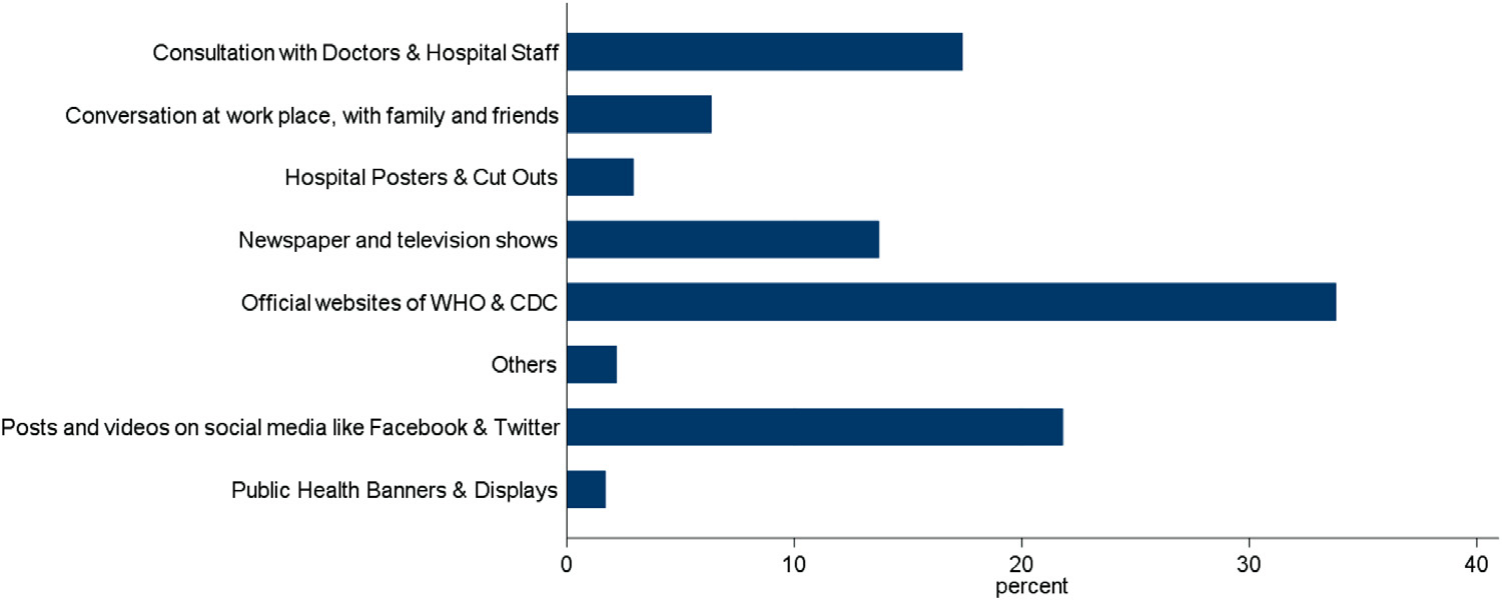
Source of information about COVID-19

The dataset gives insightful information about coronavirus-related knowledge, attitude, practices, risk perceptions, and psychological implications among Pakistani healthcare workers during COVID-19 pandemic. The COVID-19 pandemic resulted in the various psychological implications among healthcare workers due to prolonged working hours and coping with the high workload along with trauma and moral dilemmas [1]. A total of 476 responses from healthcare workers were collected during the peak season of the outbreak in Pakistan. The collected raw data used for each table was stored in a Microsoft Excel Worksheet (.xlsx) format. The dataset further divided into three primary groups: (i) sociodemographic characteristics of healthcare workers including age, gender, profession, marital status, experience, siblings (Table 1) (ii) knowledge [11 items evaluated COVID-19 related knowledge (K1-K11)], attitude [5 items perceptions being assessed about COVID-19 (A1-A5)], and practices [6 items evaluated COVID-19 related practices (P1-P6)], and (iii) psychological implications [12 items evaluated psychological disorders of healthcare workers during pandemic COVID-19 (PI1-PI12)], and their causes during COVID-19 pandemic. Besides, the knowledge variables were divided into three categories: ‘K1-K3’ three items assessed the nature of the virus, ‘K4-K9’ six items evaluated the risk of transmission of COVID-19 and ‘K10-K11’ two items assessed the possible treatment option. The detailed evaluations of responses on COVID-19-related knowledge, attitude, practice, and psychological implications among Pakistani healthcare workers were illustrated in Table 2, Table 3, Table 4. Source of information about COVID-19 was shown in Fig. 1.

**Table 1:** Sociodemographic characteristics of the healthcare workers.

Characteristics	Frequency	Percent
Age (Years)		
≤ 30 Years	336	70.6
> 30 Years	140	29.4
Gender		
Male	271	56.9
Female	205	43.1
Profession		
Doctor	202	42.4
Medical Lab Technologist	161	33.8
Nursing	64	13.4
Others	49	10.3
Marital Status		
Married	226	47.5
Single	250	52.5
Experience (Years)		
<1	93	19.5
1–5	218	45.8
6–10	79	16.6
>10	86	18.1
Siblings		
1	54	11.3
2	73	15.3
3	115	24.2
4+	234	49.2
Total	476

**Table 2:** Knowledge and attitude towards COVID-19 among healthcare workers of Pakistan.

**Questions**	I don't know, *n*(%)	No, *n*(%)	Yes, *n*(%)
**Knowledge**			
K1. Do you have previous knowledge of coronavirus?	13(2.7)	277(58.2)	186(39.1)
K2. Coronavirus is the DNA virus?	71(14.9)	351(73.7)	54(11.3)
K3. Is coronavirus structurally positive-sense RNA virus?	97(20.4)	30(6.3)	349(73.3)
K4. Does contaminated food transmit coronavirus?	39(8.2)	281(59)	156(32.8)
K5. Asymptomatic patients positive COVID-19 can infect others.	18(3.8)	12(2.5)	446(93.7)
K6. Completion of the incubation period of COVID-19 is necessary before taking a sample for the test?	53(11.1)	209(43.9)	214(45)
K7. Isolation of a suspected person is the only way to prevent COVID-19 infection?	14(2.9)	38(8.0)	424(89.1)
K8. Children are less affected because they do not have receptors for COVID-19?	121(25.4)	248(52.1)	107(22.5)
K9. Do Coronaviruses have the ability to transmit across the different species?	72(15.1)	111(23.3)	293(61.6)
K10. Currently, there is no effective cure for COVID-19, but early symptomatic and supportive treatment can help most patients recover from the infection.	22(4.6)	18(3.8)	436(91.6)
K11. Are the use of antibiotics helpful in the treatment of COVID-19 patient?	76(16)	273(57.4)	127(26.7)
**Attitude**			
A1. Do you think COVID-19 can be fully controlled?	65(13.7)	175(36.8)	236(49.6)
A2. Do you think Pakistan will win the fight against COVID-19?	56(11.8)	46(9.7)	374(78.6)
A3. Do you think imposing the lockdown is necessary to prevent the COVID-19 spreads?	22(4.6)	22(4.6)	432(90.8)
A4. Do you think you have enough PPEs to cope with this outbreak?	0(0)	343(72.1)	133(27.9)
A5. Do you ever receive any handwashing training before?	0(0)	113(23.7)	363(76.3)

**Table 3:** Practices associated with COVID-19 infection prevention.

Questions	Frequency	Percent
P1. How many times do you handshake with others in a day?		
1 time a day	31	6.5
2 times a day	28	5.9
3 or more times a day	74	15.5
Never	343	72.1
P2. How often do you wash your hands?		
1 time a day	12	2.5
2 times a day	11	2.3
3 or more times a day	448	94.1
Never	5	1.1
P3. How many times do you interact with COVID-19 patient?		
1 in day	50	10.5
1 in week	93	19.5
2 in day	31	6.5
3 or more in day	67	14.1
Never	235	49.4
P4. Do you ever educate the peoples about prevention of COVID-19 in your social circle?		
No	32	6.7
Yes	444	93.3
P5. Do you follow the complete Donning and Doffing steps for PPEs?		
No	91	19.1
Sometime	114	23.9
Yes	271	56.9
P6. Do you follow the coughing and sneezing etiquette?		
No	63	13.2
Sometime	64	13.4
Yes	349	73.3

**Table 4:** Psychological Implications of healthcare workers for the period of pandemic COVID-19.

Questions	Responses	
	Yes, *n*(%)	No, *n*(%)
PI1. Do you feel any anxiety during your job in current prevailing COVID-19 medical condition?	272(57.1)	204(42.9)
PI2. Do you feel hesitation while meeting with your family members after duty?	361(75.8)	115(24.2)
PI3. Are society member keep themselves in the distance from you, just because you are a medical professional?	265(55.7)	211(44.3)
PI4. Do you feel depressed while handling any COVID-19 patient?	284(59.7)	192(40.3)
PI5. During the duty, do you feel over-burden?	231(48.5)	245(51.5)
PI6. Do you ever abuse or shouted over any patient or his/her attendant?	108(22.7)	368(77.3)
PI7. In the current scenario, do you feel that you are not giving enough time to the patients, which are essential for them?	224(47.1)	252(52.9)
PI8. In the current situation, do you feel that your seniors have negative biases toward you?	147(30.9)	329(69.1)
PI9. In the current situation, do you feel about resigning from your current job?	67(14.1)	409(85.9)
PI10. Do you require any psychological support?	163(34.2)	313(65.8)
PI11. Deficiency of PPE's is the reason for your depression or anxiety?	307(64.5)	169(35.5)
PI12. Do you take any psychological session during this outbreak?	80(16.8)	396(83.2)

## 2 Experimental Design, Materials and Methods

To collect data, we used a descriptive online survey design to assess the knowledge, attitude, practices, and psychological implications among the Pakistani healthcare workers during COVID-19 pandemic. A questionnaire was developed, which included different questions, comprising sociodemographic characteristics, knowledge, attitude, and practices. Besides, the assessment of psychological burden or workplace-related stress was evaluated separately by using different questions. The items of the questionnaire were taken from previous research on COVID-19. After pre-testing, the online link of the questionnaire was generated using Google forms and shared among potential health workers using online tools like WhatsApp, Facebook, Twitter, etc. They were requested to forward the link to other health care workers dealing with COVID-19 patients.

The knowledge, attitude and practices related questions were retrieved from the literature [2,3,6]. Similarly, the psychological implication-based items of the healthcare workers during COVID-19 pandemic were taken from the previous studies [4,5]. To assess participants knowledge towards COVID-19 used in dataset included three subcategories: nature of the virus, risk of transmission and possible treatment. Nature of the virus included the necessary knowledge about the genetic makeup of COVID-19, and it was measured with three items. Risk of transmission of COVID-19 was measured with six items. Treatment strategies of COVID-19 patients were assessed by using two items. The attitude of respondents was measured with five items which included, the impact of lockdown, availability of personal protective equipment, and safety training before this outbreak. The knowledge and attitude questions were responded based on Yes or No with an extra option “I do not know”.

Six items measured the safety practices of healthcare workers in COVID-19 outbreak by using frequency scale method including handwashing frequency, frequency of interaction with COVID-19 patients, following the donning and doffing steps and education session for the general community. Besides, twelve questions which intimated the mental health of frontline health care workers during this pandemic were measured by using the dichotomous scale (“Yes” or “No”), and explained the work-related stress, shortage of personal protective equipment's, overburden healthcare setup and need of psychological support. The sociodemographic characteristics, knowledge, attitude, practices and psychological implication of survey questions were analyzed using descriptive statistics i.e., frequencies and percentages.

## Ethics Statement

The study was approved by **ethical review committee** of Department of Life Sciences, University of Management and Technology, Lahore. After disclosing the purpose and nature of the study, informed consent was obtained from each **participant**.

## Credit Author Statement

**Muhammad Qasim:** Methodology, Data curation, Writing- Original draft preparation, Formal analysis. **Usman Ayub Awan:** Methodology, Data curation. **Muhammad Sohail Afzal:** Conceptualization, Supervision. **Muhammad Arif Nadeem Saqib:** Conceptualization, Writing- Original draft preparation, Visualization. **Shajee Siddiqui:** Visualization, Writing- Review & Editing. **Haroon Ahmed:** Conceptualization, Data curation, Visualization

## Declaration of Competing Interest

The authors declare that they have no known competing financial interests or personal relationships which have, or could be perceived to have, influenced the work reported in this article.

## Appendix Supplementary materials

Image, application 1
